# Overcoming Patient Access Barriers in Complex Conditions: Lessons from Schizophrenia for Broader Healthcare Applications

**DOI:** 10.3390/jmahp14010002

**Published:** 2025-12-23

**Authors:** Saartje Burgmans, Anne Rieper Hald, Sayagi Tina Markandaier, Nicolas Hall, Rafael Loiseau, Xandra Lie, Bregt Kappelhoff

**Affiliations:** 1 Boehringer Ingelheim, 1043 AP Amsterdam, The Netherlandsrafael.loiseau@boehringer-ingelheim.com (R.L.); xandra.lie@boehringer-ingelheim.com (X.L.); 2 Boehringer Ingelheim, 2300 København S, Denmark; anne_rieper.hald@boehringer-ingelheim.com; 3 Frontera Group, Liverpool L24 9HJ, UK; tina@frontera-group.com (S.T.M.);

**Keywords:** schizophrenia, cognitive impairment (CIAS), access to care, health technology assessment, value-based reimbursement, multidisciplinary care, complex conditions

## Abstract

Patient access to innovative care for complex conditions like schizophrenia remains limited by systemic, clinical and policy-level barriers. Cognitive impairment associated with schizophrenia (CIAS) illustrates how critical symptom domains are often overlooked despite their impact on long-term outcomes. This study examines how systemic, infrastructural and economic factors shape access to CIAS care across eight mid-sized European countries to identify shared constraints and opportunities for improvement. Semi-structured interviews were conducted with 32 healthcare professionals and 9 health policy experts. Thematic analysis identified consistent barriers across countries, including fragmented care pathways, insufficient capacity for cognitive assessment, underdeveloped community-based rehabilitation services and reimbursement structures that favour pharmacological over psychosocial interventions. Variability across countries was shaped by differences in community infrastructure, professional training and the breadth of health technology assessment perspectives applied to non-pharmacological care. Countries with stronger community infrastructure and broader reimbursement frameworks were better positioned to deliver comprehensive care. These findings highlight that structural constraints, rather than clinical uncertainty, are the primary impediments to care in complex therapeutic areas. Addressing them will require coordinated reforms that strengthen early identification, expand multidisciplinary rehabilitation capacity and align reimbursement with functional and long-term outcomes.

## 1. Introduction

Patient access to innovative treatments is essential for improving health outcomes, both for individuals and healthcare systems. However, in complex conditions, characterised by multifactorial pathophysiology, prolonged disease trajectories and diverse patient populations, patients face unique and often greater challenges in accessing novel interventions [1,2,3]. In such conditions, ensuring patient access extends beyond regulatory approval. It requires navigating challenges such as fragmented care pathways, reimbursement constraints and clinical practices that may not fully address real-world patient needs. Together, these systemic barriers slow the adoption of therapies that could meaningfully improve quality of life and long-term outcomes.

### 1.1. Key Barriers to Patient Access

**Fragmented care pathways.** The management of complex conditions, including chronic and neuropsychiatric diseases, frequently involves multiple healthcare providers across different specialities. This often results in fragmented care pathways, delaying accurate diagnosis and access to appropriate treatment [4,5,6]. Clinical management frequently remains reactive, focusing mainly on acute manifestations or more readily recognisable symptoms. As a result, important aspects of these conditions may be under-recognised or inadequately addressed [4,7]. Limited coordination between primary, secondary and specialist services further compounds these challenges, leading to inconsistent care, treatment delays and inefficient use of healthcare resources [4,6,7]. This lack of a standardised, patient-centric and holistic care model impedes the integration of innovative interventions that address the full spectrum of disease impact [5,7].

**Misalignment between clinical outcomes and patient needs.** Clinical research frequently prioritises endpoints that are measurable in controlled settings but do not necessarily reflect the complex daily living environments of patients [8]. Patients and caregivers often value long-term goals such as maintaining daily activities, social participation, independence and overall wellbeing [1,3]. However, many commonly used biomarkers fail to capture these broader functional outcomes, while others lack feasibility in real-world clinical practice [2,3].

**Limited value-based reimbursement.** Reimbursement decisions play a vital role in determining patient access. Yet, traditional health technology assessment (HTA) frameworks may not fully capture the multidimensional value of interventions in complex diseases. Outcomes such as functional and social benefits are difficult to quantify and may, therefore, be undervalued within current evaluation models. This bias toward immediate or easily measurable results can exclude or undervalue innovative interventions that provide long-term benefits to patients and communities [9,10,11].

**Clinical practice and implementation gaps.** Even when treatments are approved and reimbursed, their uptake in clinical practices can be slow, particularly in complex disease areas [12]. Clinical routines often reflect historical standards of care, and integrating new approaches requires time, education and compelling evidence. Healthcare professionals (HCPs) can be cautious about adopting new interventions, especially when diagnostic uncertainty exists or when scientific experience with a new therapy is limited [12,13,14].

### 1.2. Schizophrenia as a Case Study of Structural Barriers

Schizophrenia exemplifies how these systemic challenges manifest in practice. Despite substantial advances in understanding the disorder, individuals with schizophrenia continue to face barriers in diagnosis, treatment and continuity of care. Diagnosis is often delayed until the onset of acute psychotic episodes, when positive symptoms like hallucinations or delusions become apparent. However, negative and cognitive symptoms—such as anhedonia, social withdrawal and impaired attention—typically precede psychosis and persist throughout the course of disease [15]. These symptoms are prevalent: approximately 40% of patients experience prominent negative symptoms, and up to 98% demonstrate some degree of cognitive impairment [16,17]. These additional challenges often continue beyond acute psychotic episodes and increase the risk of treatment resistance and nonadherence, worsen clinical outcomes and impair overall functioning, proving to be a critical limiting factor in schizophrenia recovery [16,17,18,19].

Cognitive impairment associated with schizophrenia (CIAS) affects multiple cognitive domains, including memory, attention, problem-solving and social cognition [20,21]. These impairments are a key driver of long-term functional difficulties, affecting individuals’ capacity to sustain employment, live independently and participate in society. CIAS also represents a major contributor to the overall societal and economic burden of schizophrenia [16,17,18]. Even modest improvements in cognitive performance have been associated with better functioning and quality of life [18,21].

Despite these insights, real-world implementation of CIAS assessment and treatment remains limited. The European Psychiatric Association (EPA) recommends a combination of pharmacological and psychosocial interventions to improve cognitive outcomes; however, translation of these guidelines into practice has been slow [18]. The reasons for this science-to-service gap are only now beginning to be defined, but current evidence suggests they include a range of interrelated financial, structural and systemic factors [17,18].

### 1.3. Key Barriers to Patient Access in Schizophrenia

**No standardisation of diagnosis and treatment.** The diagnosis and quantification of CIAS remain inconsistently applied across clinical settings. Although the EPA recommends the routine assessment of CIAS, such assessments are not commonly implemented in practice. Existing diagnostic batteries are often considered too time-consuming or impractical for routine clinical use [20,21]. This has led to considerable variability in the recognition and management of CIAS across Europe [20]. The absence of a unified diagnostic framework, coupled with limited treatment options, further complicates clinical decision-making and hinders effective care [18].

**Fragmented care pathways.** The management of CIAS is frequently impeded by fragmented or siloed healthcare systems [15,22]. Schizophrenia care often focuses on positive symptoms, while the interconnected nature of cognitive, negative and social symptoms receives less systematic attention, despite evidence that integrated, multidisciplinary approaches lead to better outcomes [15,22]. For example, cognitive remediation therapy is more effective when combined with structured psychosocial rehabilitation strategies such as exercise or occupational therapy [18,22]. However, these multidisciplinary models remain underdeveloped and inconsistently applied across European healthcare systems [22].

**Misalignment between clinical outcomes and patient needs.** While pharmacological treatments are effective for psychotic episodes, they provide limited benefit for CIAS and, in some cases, may exacerbate cognitive deficits [21,22]. Interventions that support cognitive functioning are rarely implemented, even though cognitive impairment has substantial real-world consequences for social and occupational performance [22,23]. Moreover, patient awareness of cognitive symptoms is often limited, resulting in under-reporting during clinical consultations [17].

**Limited value-based reimbursement.** Although European clinical guidelines recommend integrating psychosocial interventions such as social skills training and cognitive behavioural therapy alongside pharmacological treatment, reimbursement systems typically do not support these approaches [18]. This gap is largely due to the limited availability of real-world evidence demonstrating measurable functional improvements, making it harder to demonstrate cost offsets [18,22]. Consequently, interventions that produce gradual or indirect improvements, such as social functioning and reintegration into working life, are undervalued in traditional reimbursement frameworks [18]. Even interventions with clinical evidence, like cognitive remediation therapy, face significant implementation barriers due to limited qualified personnel and insufficient healthcare funding [22].

**Clinical practice and implementation gaps.** Despite increasing recognition of the need to integrate CIAS assessment and management into standard schizophrenia care, implementation remains inconsistent [15,22]. Lack of clinician training and resource limitations all contribute to underdiagnosis, and cognitive symptoms are frequently misattributed to medication side effects rather than the illness itself [17,21]. The absence of effective screening tools and limited availability of approved pharmacological or psychosocial interventions further contribute to clinical inertia, reinforcing the perception that cognitive deficits are either untreatable or secondary in importance [18,21].

### 1.4. Study Rationale and Objectives

Despite growing recognition of the importance of CIAS, significant gaps remain between research, policy recommendations and real-world implementation. Our review of the existing literature identified a limited and uneven evidence base on the recognition, assessment and management of CIAS in Europe. Most available publications originate from the United States or the larger ‘EU5’ countries, with only sparse data from the mid-sized European health systems. Therefore, how barriers to CIAS care manifest within mid-sized European contexts, and how health system structures shape them, remain insufficiently understood.

To address this gap, the present study uses qualitative research conducted across eight mid-sized European countries to understand how systemic, infrastructural and economic factors influence the assessment and management of CIAS. This approach enables the identification of shared patterns and system-level constraints, offering insights that may inform the design of more effective access strategies for CIAS as well as other complex conditions with similar systemic challenges.

## 2. Materials and Methods

### 2.1. Study Design

This was a cross-sectional study comprising semi-structured qualitative interviews that were conducted with HCPs (*n* = 32) and health policy experts (HPEs; *n* = 9) in the Netherlands, Belgium, Denmark, Sweden, Norway, Finland, Portugal and Greece. These countries were selected to reflect the distinctive characteristics of mid-sized countries, which are less frequently explored compared to the US and ‘EU5’ countries (France, Germany, Italy, Spain and the United Kingdom). Interviews aimed to validate current care pathways and delivery systems for people living with schizophrenia, examine how healthcare structures influence CIAS adoption and explore barriers to patient access to cognitive interventions. Interview guides are included in the Supplementary File.

### 2.2. Recruitment and Sampling

HCP participants were recruited via a third-party recruitment agency using its internal database, while HPE participants were recruited directly by an independent research agency through email and social media outreach. All participants provided written informed consent and were compensated at fair market value.

Participants were purposively sampled according to predefined inclusion criteria (Table S1 in the Supplementary File). HCPs were required to have sufficient experience in schizophrenia management and with varying familiarity with CIAS. HPE participants were required to have sufficient experience within healthcare and reimbursement for severe mental illness and were not employed as public officials at the time of the study.

The target sample in each country was six HCPs (three psychiatrists, one psychologist, one psychiatric nurse, one general practitioner) and two HPEs. Although this quota was not met in all countries, thematic saturation was assessed in real time using Rapid Research, Evaluation and Appraisal (RREAL) sheets [24]. Saturation was considered achieved when no new themes or substantive insights emerged from subsequent interviews. This was reached in all countries except Greece, where fewer interviews were conducted (see Section 5).

### 2.3. Data Collection

Interviews were conducted between February and December 2024 by experienced qualitative researchers trained in the study aims (NH, CM, AP, JB). Three semi-structured discussion guides were used (HCP-psychiatrist; HCP-other; HPE) to explore patient access barriers, care pathway structures, reimbursement challenges and implementation issues. Guides were developed based on insights from the literature review and adapted for each participant group.

Interviews lasted approximately 60 min and were conducted in English via Microsoft Teams. All interviews were audio-recorded and transcribed verbatim. Transcripts were de-identified to ensure participant confidentiality.

### 2.4. Data Analysis

Data analysis followed a thematic analytic framework combining deductive and inductive approaches [25]. The initial interview guides were developed deductively from findings in the literature review and further refined inductively based on emerging insights from the interviews.

RREAL sheets, table-based matrices designed to capture key themes in real time, were used throughout data collection. As interviews progressed, the sheets were updated iteratively to capture emergent themes and monitor data saturation. This approach provided a consistent structure and allowed systematic comparison of findings across countries, participant groups (HCPs vs. HPEs) and HCP specialties (e.g., psychiatrist vs. psychologist). Transcripts were then coded thematically with qualitative analysis software (Dovetail).

To enhance the reliability and validity of the research findings, several methodological strategies were implemented. All interviewers received detailed training on the study objectives, interview procedures and analytic framework to ensure consistency in data collection and interpretation. The use of RREAL sheets provided a transparent and standardised process for documenting and comparing findings across countries and participant groups in real time, helping to minimise researcher bias and enhance dependability.

An initial a priori codebook was developed based on the literature review and then refined iteratively throughout data collection and analysis. This codebook provided a structured framework for coding, helped ensure consistency across researchers and guided the development of themes. Coding and theme development were reviewed regularly through team discussions, with any discrepancies resolved by consensus to maintain consistency and reproducibility across the dataset.

Validity was strengthened through multiple complementary strategies. An audit trail was maintained to document all key analytical decisions, supporting transparency and confirmability of results. Data triangulation was applied by comparing insights from the literature review with interview findings to validate interpretations and ensure the conclusions were supported by both existing evidence and participant perspectives. Credibility was further reinforced through iterative analysis, which allowed the research team to refine emerging themes and ensure that interpretations accurately reflected participant views. Finally, reflexive practice was maintained throughout data collection and analysis to increase awareness of potential researcher bias and to safeguard the authenticity of the findings.

## 3. Results

Interviews were conducted with 32 HCPs and 9 HPEs across the eight countries. Sociodemographic and clinical characteristics for HCPs are summarised in Table S2 in the Supplementary File. HPE participants had a mean of 22 years of experience within the health sector (range 4–35 years), with professional backgrounds spanning academia and research (*n* = 5), the pharmaceutical industry (*n* = 3), government or public organisations (*n* = 2) and consultancy (*n* = 1).

By systematically applying the RREAL approach, accounts from HCPs were synthesised to construct a typical care pathway for people living with schizophrenia across these mid-sized European countries. This approach allowed us to capture a diverse range of professional perspectives while identifying common institutional, infrastructural and economic barriers across healthcare systems. Insights from HCP and HPE participants also informed the development of a comparative framework, categorising countries into two healthcare system archetypes based on their degree of deinstitutionalisation and integration of community care.

### 3.1. Typical Care Pathway

Analysis of HCP interviews revealed a broadly consistent care pathway for people living with schizophrenia, structured around four key stages: acute treatment, stabilisation and diagnosis, intensive follow-up and long-term follow-up. Across all countries, participants identified common barriers, despite significant differences in healthcare organisation (explored further in the Section 3.2).

**Figure 1:**
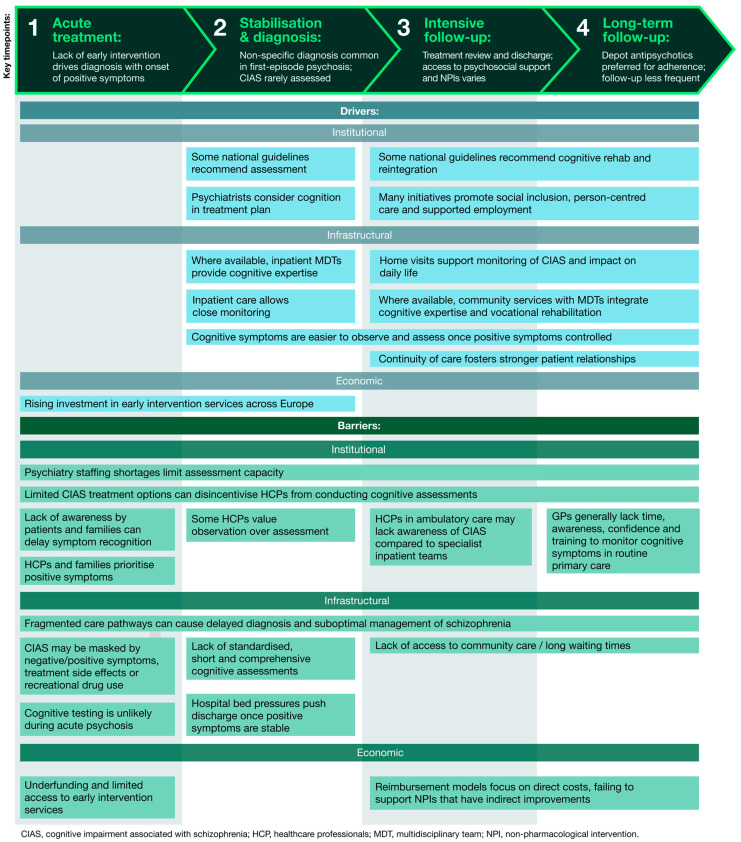
Drivers and barriers to cognitive care along a typical treatment pathway for people living with schizophrenia. The figure presents a generalised patient journey across four key stages of care: acute treatment, stabilisation and diagnosis, intensive follow-up and long-term follow-up (shown at the top). For each stage, the figure highlights the main drivers and barriers, derived from thematic analysis of interviews with HCPs and HPEs. Together, these layers illustrate how institutional, infrastructural and economic factors interact throughout the care pathway, influencing the assessment and management of CIAS.

Figure 1 summarises these common barriers and drivers across all countries, grouped into institutional, infrastructural and economic challenges. It is directly informed by thematic analysis of HCP and HPE interviews, showing how these factors interact at each stage of the care pathway to influence the assessment and management of CIAS.

**1. Acute treatment.** HCPs reported a general lack of early intervention in psychosis services, resulting in missed opportunities for timely identification of the prodromal phase of schizophrenia, characterised by negative and cognitive symptoms. Instead, patients typically enter the system through emergency services at a later stage with acute positive symptoms of psychosis.

Following triage, most patients are subsequently admitted to a psychiatric hospital or the psychiatric department of a general hospital for short-term management. At this stage, which typically lasts only a few weeks, management focuses primarily on controlling positive symptoms.

*“Cognitive symptoms and negative symptoms are much more dangerous than positive symptoms, which are only the top of the iceberg. Positive symptoms are what you see, but the real problem is under the water.”*

Psychiatrist, Finland

**2. Stabilisation and diagnosis.** While some patients may be diagnosed with schizophrenia during an inpatient admission, many receive an unspecified psychosis classification instead, particularly if it is their first episode of psychosis. HCPs noted that psychiatrists often delay a formal schizophrenia diagnosis due to uncertainty and stigma concerns.

Participants reported a strong awareness of CIAS among multidisciplinary psychiatric teams working in inpatient settings. Although such impairments are noted during clinical observations, formal cognitive assessments remain uncommon due to unclear guidelines, lack of standardised tools and limited treatment options. Some also questioned the usefulness of measuring CIAS when there are few effective treatment options available.

When cognitive testing does occur, it typically is a part of a much larger battery of psychological testing that can take several hours to complete. HCPs generally viewed this process as burdensome and time-intensive, particularly given the limited availability of psychologists. As a result, most people living with schizophrenia do not undergo any cognitive testing.

*“Good tests take sometimes two or three hours, so they are long. And of course, psychologists are also expensive and there are not enough psychologists to do it.”*

Psychiatrist, the Netherlands

**3. Intensive follow-up.** After stabilisation of acute positive symptoms, patients are typically discharged to ambulatory or community services, with little to no treatment provided for negative or cognitive symptoms. Psychosocial interventions are sometimes offered to patients alongside ongoing antipsychotic treatment; however, the levels of access to these programmes differ considerably between and within countries. There is also variable provision of other non-pharmacological interventions designed to help patients’ social and functional recovery, including rehabilitation therapy and reintegration support.

*“I think these programmes that are really made to have cognitive remediation should be more accessible.”*

Psychiatrist, Belgium

**4. Long-term follow-up.** Patients may be followed up in outpatient clinics or at home by community teams. Long-term management primarily involves the continued use of antipsychotic medications, often through depot injections due to their perceived benefits in supporting treatment adherence.

*“If it can be an injectable, if it can be a depot, definitely. Because, you see, the patients have to be quite well to take tablets every day.”*

Psychiatrist, Norway

HCPs noted that follow-up becomes less frequent over time as the focus shifts to longer-term monitoring of treatment adherence and ongoing symptom control. Appointments may eventually be spaced out to every six to twelve months and take place in outpatient settings, general practice surgeries or within the community.

### 3.2. Country Archetypes

**Figure 2:**
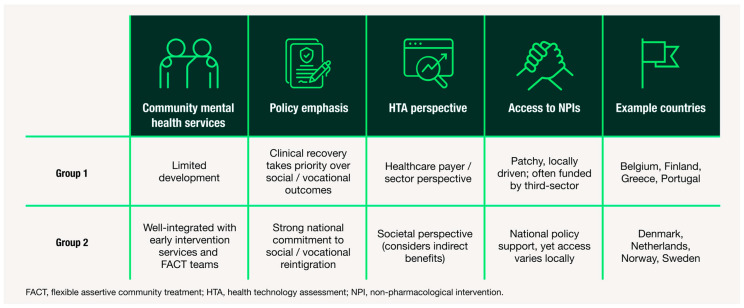
Summary of country archetypes based on thematic insights from HCP and HPE interviews. Two archetypes of mid-sized European healthcare systems for schizophrenia care are shown, based on differences in: (1) availability of community mental health services, (2) policy emphasis on social reintegration, (3) HTA perspective and (4) access to non-pharmacological interventions. Example countries are provided for each archetype. This framework highlights system-level differences in support for CIAS and identifies potential targets for reform.

To examine variation between healthcare systems, we then conducted a comparative analysis of country-level patterns. Given the small sample size, countries were grouped into two archetypes to capture patterns in system-level characteristics while preserving the ability to highlight differences between healthcare systems. These archetypes are defined by the extent of deinstitutionalisation—including both the diversion of hospital-based services and the development of community-based alternatives—as well as their attitudes towards the holistic reintegration of individuals into society. While all countries are trending toward greater emphasis on community care and social reintegration, the two archetypes reflect differing rates of progress and approach. Figure 2 summarises these findings, illustrating how structural and policy-level features vary across archetypes and how these differences may influence CIAS care. By comparing archetypes, we can distinguish commonalities in care pathways from country-specific variations, clarifying both shared challenges and differences across healthcare systems.

### 3.3. Deinstitutionalisation and Community Care

While most countries have made significant progress in diverting patients with serious mental illness away from hospital settings, a key difference between Group 1 and 2 archetypes is the availability of community care. Participants from Group 1 countries described people living with schizophrenia as being affected by a gap created by hospital downsizing without parallel expansion of community support. In these settings, community mental health teams are often underdeveloped or under-resourced.

*“We have not very good teams outside of the hospital. That’s a problem.”*

Psychiatrist, Belgium

By contrast, participants from Group 2 described well-established community infrastructures for people with severe mental illness that support continuity of care. In particular, flexible assertive community treatment (FACT) teams were frequently cited as essential for managing long-term consequences of schizophrenia. These outreach teams can adapt the intensity of support according to the patients’ needs, providing home-based care during acute phases and more routine follow-up during stability.

*“It’s a decision by the state that [FACT] should be the way we treat the longer-term psychotic illnesses because the old way of booking and waiting for patients to show up doesn’t work, not when you’re dealing with psychosis. You need to be assertive, you need to be flexible, and you need to be able to treat on home visits and to meet your patients within society and not sit and wait for the patient coming to you at your clinic.”*

Psychiatrist, Denmark

The presence of such integrated, community-based services acts as a system-level enabler of cognitive management. Consequently, Group 2 countries are generally better positioned for CIAS care adoption due to their established outreach and multidisciplinary models.

### 3.4. Rehabilitation and Reintegration

Another key distinction between the groups relates to the societal perspectives on non-pharmacological interventions for people living with schizophrenia. In Group 1 countries, interventions aimed at cognitive remediation, vocational rehabilitation or social skills training are less accessible. Participants described fragmented service provision, with limited national coordination and heavy reliance on local or on third-sector initiatives such as non-profit organisations.

*“The main problem is the public system doesn’t have many rehabilitation units to improve their cognitive [symptoms], specifically for schizophrenia.”*

Psychiatrist, Portugal

In Group 2 countries, rehabilitation and reintegration are more systematically incorporated into national strategies for mental health. These countries place stronger emphasis on person-centred recovery, supported employment and social inclusion. Many FACT teams in these countries include dedicated staff focused on vocational rehabilitation and daily functioning. Although regional variation persists, these services are generally more widespread and accessible.

*“The social skills training within the FACT or even the supported employment, it’s very important, I mean, very important if they can have daily routine and stuff like that.”*

Psychiatrist, Sweden

### 3.5. Funding and Resources

Differences in funding priorities and value assessment frameworks were also evident between the two groups. HPEs described Group 1 as predominantly payer-driven, with cost-effectiveness analyses focused narrowly on healthcare expenditure. This approach tends to overlook indirect benefits of cognitive treatments, such as improved patient productivity and reduced caregiver burden, in formal value assessments.

In addition to having a higher overall investment in mental health infrastructure, Group 2 tend to apply a societal perspective in their HTA evaluations, although to varying degrees (greater in the Netherlands and Sweden; lesser in Denmark and Norway). This broader perspective is viewed as advantageous for the adoption of CIAS care pathways, as it recognises indirect benefits of improved cognition beyond healthcare.

*“The societal cost can be included in the Netherlands. In Belgium, they don’t take it into account.”*

Health Policy Expert, Belgium

Nevertheless, even in better-resourced Group 2 settings, implementation challenges remain. Support for non-pharmacological interventions often depends on local administrative procedures, funding availability and regional policy priorities, despite approval at the national level. As a result, discrepancies in service availability persist across all countries, leading to uneven access, delays in care and variable quality of provision.

## 4. Discussion

This study provides one of the first cross-country qualitative analyses of access barriers to CIAS care across eight mid-sized European countries. By comparing perspectives from HCPs and HPEs, the study identifies both common systemic constraints and context-specific barriers that shape access to cognitive care. Additionally, the study introduces an archetype framework that classifies these mid-sized European healthcare systems based on the degree of deinstitutionalisation and approaches to social reintegration, providing a new model for understanding cross-country differences in CIAS care. While many of the findings align with previous literature, this research contributes new, empirically grounded insights into how and why these barriers persist, where opportunities for structural change lie and offers transferable lessons that can inform access strategies for other complex conditions characterised by similar systemic challenges.

Across all eight countries, the study found that current healthcare systems are not sufficiently structured to manage the full complexity of schizophrenia, particularly the cognitive and negative dimensions of the condition. Although the persistence of these care gaps is well documented [17,21,22], this study expands the evidence base by demonstrating that such challenges extend beyond large or well-studied health systems (such as the US and EU5) and remain pervasive even in mid-sized European countries with varying healthcare structures and resources.

A key insight emerging from this study is that barriers to CIAS assessment are not primarily driven by a lack of clinical awareness or education. Most clinicians across settings recognised the significance of cognitive impairment and its impact on patient recovery. Instead, barriers were structural and systemic, including time constraints and workforce shortages. The absence of validated, short-form cognitive assessments suitable for use by non-specialist providers, such as psychiatric nurses or occupational therapists, was repeatedly cited as a practical barrier to implementation.

Furthermore, this study provides new comparative evidence on how financing structures and reimbursement mechanisms influence the availability of non-pharmacological interventions. While psychosocial and cognitive remediation therapies are recognised as essential components of comprehensive care, they are not consistently reimbursed across countries, even in those with otherwise centralised or well-resourced systems. In the Netherlands and Sweden, for example, HTA frameworks formally allow the consideration of broader societal benefits—such as caregiver quality of life, productivity losses and cross-sectoral costs—in economic evaluations [26,27]. Yet even in these settings, methodological challenges persist, such as how to best measure and value productivity effects and caregiver burden [28]. As a result, a persistent policy misalignment remains, with non-pharmacological interventions continuing to be underfunded despite their recognised contribution to functional recovery.

Taken together, these findings suggest that while awareness of CIAS is improving, translation into practice is constrained by system-level gaps, including the absence of national mandates for cognitive assessment, limited investment in rehabilitation infrastructure and reimbursement that prioritises pharmacological over psychosocial interventions.

These challenges are not unique to schizophrenia; rather, they reflect a wider systemic issue in how healthcare systems respond to complex, multidimensional conditions. In areas such as oncology and metabolic conditions, care delivery is frequently compartmentalised with a focus on clinical or biomarker-driven outcomes. This focus, combined with persistent underinvestment in non-pharmacological interventions, can lead to missed opportunities for better patient outcomes.

### 4.1. Systemic Gaps in Treating Other Mental Health Conditions

Similar systemic barriers are seen across other complex mental health conditions. Cognitive impairment, although prevalent across neuropsychiatric conditions, remains under-recognised even when it is a core feature, such as in major depressive disorder [29,30]. Despite its strong link with social and occupational functioning, routine cognitive assessment and availability of targeted pharmacological treatments remain limited [30]. These gaps extend beyond cognitive domains: more than 70% of people worldwide who need mental healthcare do not receive appropriate services [31]. Persistent barriers to timely care include stigma, poor symptom recognition, inadequate interdisciplinary coordination, resource deficits, limited clinician training and underdeveloped mental health infrastructure [31,32,33].

Mental health-specific barriers are also evident across a range of chronic conditions. For instance, psychological distress often goes unrecognised throughout the oncology care continuum [34,35,36]. Similarly, in cardiovascular disease, recommended screening for mental disorders is inconsistently applied [37].

These examples underscore the broader systemic need for integrated care models that combine physical and psychological support. Progress requires greater investment in multidisciplinary training, routine neurological and psychological assessment and clear policy frameworks that make psychosocial care a standard part of medical practice.

### 4.2. Insufficient Progress in Supporting Real-World Functional Recovery

Across many medical specialties, there is a consistent disconnect between clinical management and patient-centred functional outcomes. In oncology, major therapeutic advances have improved survival rates, but long-term functioning and quality of life are still overlooked. Traditional clinical assessments often fail to reflect the full impact of cancer and its treatment on the patients’ daily lives [38]. Patient-reported outcomes (PROs) can provide valuable insight into wellbeing, yet they are not routinely used in oncology, with only about one in four practitioners incorporating them into regular practice [38,39,40]. Barriers include time constraints, uncertainty about how to interpret PRO data and difficulties integrating these tools into existing workflows [38,41].

A similar issue arises in pain management. Pain intensity is routinely measured, but broader effects, such as emotional distress or social disengagement, are rarely evaluated [42,43]. Although experts have called for greater use of digital-friendly, multidimensional PRO tools, adoption remains low due to the absence of standardised, validated instruments [42,44].

Taken together, these patterns reveal a sustained misalignment between clinical metrics and patient priorities, emphasising the need for systemic reforms that position functional recovery as a primary treatment objective.

### 4.3. Lack of Coordinated, Holistic Care for Patients with Multifaceted Needs

The fragmentation seen in schizophrenia care also affects the management of other complex, multisystem conditions such as metabolic disease and pulmonary fibrosis. In metabolic conditions—including obesity, type 2 diabetes, chronic kidney disease and metabolic dysfunction-associated steatohepatitis (MASH)—clinical practice often centres on specific biomarker targets, like HbA1c or LDL-C [45,46,47]. This narrow focus can overlook broader health determinants, like long-term cardiovascular risk, comorbidities and lifestyle factors [45,48]. Although guidelines recommend multidisciplinary management, coordination across specialties remains limited in real-world practice [49,50]. For instance, while over one-third of people living with type 2 diabetes or obesity may have MASH, the condition is undiagnosed in up to 88% of cases [51,52,53].

A similar disconnect exists in rheumatoid arthritis-associated interstitial lung disease (RA-ILD). Rheumatoid arthritis is a multisystem condition that is predominantly managed within rheumatology, yet pulmonary manifestations often go unrecognised [54,55,56]. This occurs because of non-specific symptoms (e.g., cough, dyspnoea or fatigue), lack of integrated diagnostics and limited awareness among non-specialists [57,58]. Care is further impeded by inconsistent multidisciplinary coordination and hesitancy to initiate anti-fibrotic treatment [57,59].

These cross-disciplinary parallels show that many healthcare systems still rely on reactive, disease-specific models. To meet the multidimensional needs of patients with complex conditions, system-level integration is needed.

### 4.4. Recommendations for Future Directions

**Figure 3:**
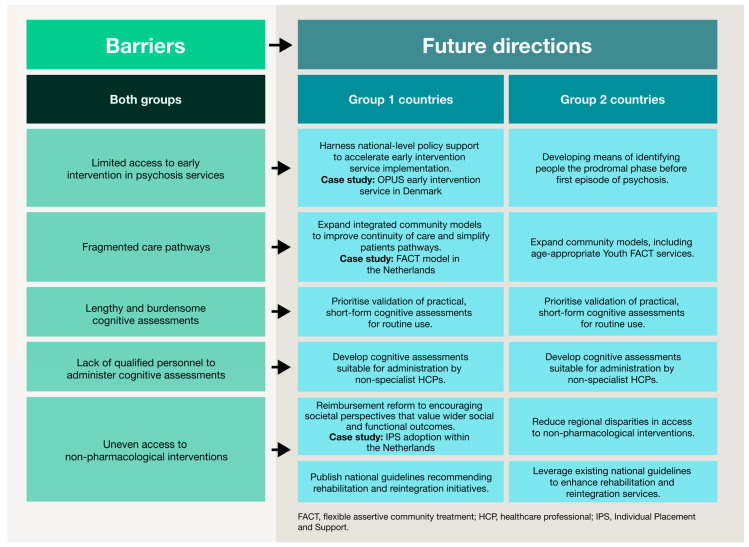
Barriers to CIAS care and future directions stratified by country archetype. This figure summarises high-level obstacles across the schizophrenia care pathway. Future directions are presented according to the two country archetypes, illustrating tailored strategies to improve service provision, expand community-based support, strengthen multidisciplinary approaches and enhance reimbursement and policy mechanisms. This visual framework illustrates how tailored interventions can support functional and patient-centred outcomes in different healthcare contexts.

The challenges identified in this study reflect gaps that extend across healthcare systems, beyond schizophrenia alone. The following directions are therefore relevant to other complex conditions that require coordinated, multidisciplinary care. Core themes from this research—such as early intervention, cognitive and functional recovery, community-based support and structural reform—should guide efforts to create more responsive, equitable and patient-centric health systems. Figure 3 summarises these future directions for schizophrenia based on country archetype.

**Prioritising early identification.** In schizophrenia, early intervention services are proven to improve clinical and functional outcomes by reducing the duration of untreated psychosis [60,61,62]. This need for timely, proactive intervention is mirrored in other therapeutic areas. For example, MASH is often diagnosed only at advanced stages, even though earlier identification can prevent progression to cirrhosis or cancer [63,64]. Similar gaps persist in cardiovascular disease, oncology and RA-ILD, reinforcing the need to integrate early detection into routine care pathways [65,66].

Despite strong evidence for their effectiveness and cost-efficiency [67,68], early intervention in psychosis services remain inconsistently implemented across Europe [69]. Denmark’s OPUS programme shows that rapid implementation of early intervention services is possible when supported by clear policy, sufficient funding and structured service frameworks [62,70,71]. Similarly, the introduction of national cancer patient pathways in Scandinavian countries [72] shows that early detection and treatment can be scaled effectively with government commitment and appropriate training for HCPs. Together, these examples indicate that proactive approaches require a combination of policy support, structured pathways and investment in staff education and capacity.

**Embedding multidisciplinary care.** Our findings highlight the need to make cognitive assessment and treatment a standard part of schizophrenia management. Across other therapeutic areas, multidisciplinary approaches are also increasingly being recognised as essential. For instance, integrated models for chronic kidney disease and other metabolic conditions help delay disease progression and reduce reliance on invasive treatments [50,73,74]. In many conditions, such as cancer, physical and functional wellbeing are strong predictors of long-term outcomes but are often neglected [75,76].

The FACT model, used widely in Group 2 countries (the Netherlands, Norway, Sweden and Denmark), demonstrates how an integrated, community-based service can improve continuity of care. FACT teams foster collaboration across multiple settings, provide home-based support and link patients to community resources [77,78]. Comparable approaches, such as integrating palliative and psychological care throughout the oncology patient pathway, show benefits to survival, symptom control, decision-making and quality of life [34,79]. These examples illustrate the importance of embedding multidisciplinary models to reduce the fragmentation of care and improve outcomes in complex diseases.

**Advancing patient-centred recovery.** Beyond achieving clinical remission, management should focus on personal and functional recovery. This creates opportunities for shared decision-making, where the individual’s priorities, values and unique circumstances help shape the care plan [80]. Tools such as patient-reported outcome measures (PROMs) and cognitive assessment batteries capture patients’ lived experiences, but practical use is limited by workflow and technological barriers [20,38,39,42]. Future care models must prioritise short, digital-friendly assessments that integrate into electronic health records, provide automated data feedback and include training for HCPs to interpret and act on results.

Novel cognitive assessments and non-pharmacological interventions have been designed to reduce these practical barriers by addressing factors such as participant and assessor burden, the feasibility of remote and computerised completion, availability of training and the usability by non-specialists [81,82,83]. Overcoming these practical barriers can enhance the feasibility of using assessments and interventions in everyday clinical practice. In turn, this can facilitate patient-centred care by informing prognosis, enabling personalised treatment and ultimately improving patient outcomes [84].

**Expanding community-based and peer-driven support systems.** Community-based services, which centre on continuity, recovery and real-life functioning, counter the “revolving door” effect often seen in acute, hospital-centric models [85]. Findings from the qualitative interviews align with growing recognition in other domains that structured community and peer-to-peer models (e.g., Clubhouse International, Alcoholics Anonymous and cancer support networks) provide enduring psychosocial benefits [86,87,88]. Scaling these programmes within formal healthcare systems can improve patient engagement, adherence and long-term outcomes. Effective expansion requires clear funding streams, standardised quality frameworks, integration of peer specialists into multidisciplinary teams and digital platforms to facilitate communication and monitoring.

**Reforming reimbursement and policy to support holistic care.** Structural changes, especially in reimbursement, are also needed to enable sustained investment in comprehensive, patient-centric care. While pharmacological therapies typically have clear reimbursement pathways, access to non-pharmacological interventions remains limited [89]. This issue may be compounded by the need for robust evidence demonstrating effectiveness and cost-efficiency, which is required for coverage decisions [90,91]. HTAs should evolve to consider functional and societal impacts, including quality of life, employment, independence and reduced caregiver burden, as they are critical metrics in chronic disease management. Examples such as the integration of individual placement and support, a vocational rehabilitation programme for people with severe mental illness, into Dutch health insurance funding illustrates how evidence-based interventions addressing societal and functional outcomes can secure reimbursement and policy support [92]. These lessons highlight the importance of combining research, real-world outcomes as well as funding and policy mechanisms to make holistic, patient-centred management widely accessible.

### 4.5. Tailoring Approaches by Country Archetype

Although recommendations overlap across countries, the archetypes identified in this study suggest the need for country-specific access strategies that reflect each health system’s structure, policy priorities and reimbursement mechanisms. Group 1 countries, which are at an earlier stage of implementing integrated mental healthcare, should prioritise the gradual expansion of multidisciplinary care models such as those established in Group 2 countries. This includes developing community-based services, improving coordination between primary and specialist services and increasing training for non-specialist providers to identify and manage cognitive impairment. However, limited healthcare budgets remain a major barrier, particularly for low- and middle-income countries [61].

In Group 2 countries, where care is much more established, future priorities should focus on closing existing gaps and expanding the reach of services to underserved populations. Efforts should include strengthening reimbursement mechanisms and scaling up non-pharmacological interventions that can support holistic, functional recovery. At the same time, these countries can invest in targeted areas of expansion and specialisation. For example, the recent development of Youth FACT teams in the Netherlands—tailored to young people up to 24 years old—illustrates how services can be adapted to meet the specific needs of different population groups [93]. This approach parallels developments in oncology, where dedicated teenage and young adult (TYA) cancer wards in England deliver age-appropriate care that addresses both medical and psychosocial needs [94].

## 5. Limitations

This study has several limitations that should be considered when interpreting the findings. First, the targeted sampling quota of six HCPs and two HPEs was not achieved across all participating countries. In particular, Greece, Sweden and Finland had fewer participants than expected, with Greece especially limited in participant numbers due to recruitment issues. This smaller and uneven sample size may limit the generalisability and comparative strength of insights across regions.

Second, while purposive sampling was used to recruit participants with relevant expertise in CIAS, this approach may still introduce selection bias. Participants with a particular interest or more extensive experience in CIAS were possibly more motivated to take part, which could lead to overrepresentation of more specialised viewpoints. However, the consistency between interview insights and the findings of the accompanying literature review provides some reassurance of validity and reduces concern that the sample skewed results.

While the study aimed to ensure broader cross-country coverage, differences in healthcare structure, funding mechanisms and levels of deinstitutionalisation across the eight countries introduce contextual heterogeneity. These differences may have limited the ability to draw direct one-to-one comparisons between national systems. Nevertheless, the use of the country archetype framework helped group countries with broadly similar health system characteristics, improving the interpretability and transferability of findings across contexts.

Language differences also presented potential challenges. Interviews were conducted in English, which may have minimised language-related variation in interpretation. However, for participants for whom English is not the first language, nuances in expression or emphasis may have been less precise, particularly when discussing complex clinical or policy issues.

Finally, the absence of patient and caregiver perspectives represents an important limitation. Although it was beyond the scope of the current study, prior initiatives from Boehringer Ingelheim have documented key priorities expressed by patient organisations and advocacy groups. These needs include greater education and awareness, improved access to peer and expert support, more robust training for caregivers, stronger involvement in research and more effort to reduce stigma. Future research should incorporate direct input from these groups to ensure that care models are aligned with lived experiences and real-world recovery goals.

## 6. Conclusions

Despite advances in understanding the pathophysiology and treatment of schizophrenia, patients continue to face barriers to care, particularly in the recognition and management of cognitive impairment. Through a cross-country qualitative analysis, this study identifies a set of common institutional, infrastructural and economic challenges that impede CIAS care, ranging from limited capacity for cognitive assessment to reimbursement structures that prioritise pharmacological treatment. At the same time, the interviews revealed important country-specific differences shaped by broader system characteristics.

To interpret these variations, we developed a country archetype framework that captures how differing degrees of deinstitutionalisation, community capacity and policy emphasis on social reintegration influence the care pathway. This framework clarifies why countries with otherwise similar resources diverge in their ability to implement cognitive assessment and provide rehabilitation services.

By identifying both shared and context-specific barriers, this study advances understanding of how system-level constraints shape access to CIAS care. These findings can inform targeted policy reforms, including strengthening training, expanding reimbursement to cover non-pharmacological interventions and investing in early intervention and community-based recovery infrastructure. More broadly, the structural barriers identified here mirror challenges observed in other complex conditions, suggesting that addressing them could improve care delivery across multidisciplinary and chronic disease pathways.

## Data Availability

This study generated research data that are not publicly available. Data supporting the findings are available from the corresponding author upon reasonable request.

## Supplementary Materials

The following supporting information can be downloaded at: https://www.mdpi.com/article/10.3390/jmahp14010002/s1. Supplementary File S1: Interview discussion guide for Psychiatrists. Supplementary File S2: Interview discussion guide for other HCPs. Supplementary File S3: Interview discussion guide for HPEs. Supplementary Table S1: Participant inclusion criteria. Supplemental Table S2: Sociodemographic (A) and clinical (B) characteristics of HCP participants (*n* = 32).

## Abbreviations

The following abbreviations are used in this manuscript:

CIAS	Cognitive impairment associated with schizophrenia
EPA	European Psychiatric Association
FACT	Flexible assertive community treatment
HbA1c	Haemoglobin A1c
HCP	Healthcare professional
HPE	Health policy expert
HTA	Health technology assessment
MASH	Metabolic dysfunction-associated steatohepatitis
LDL-C	Low-density lipoprotein cholesterol
PRO	Patient-reported outcome
PROM	Patient-reported outcome measure
RA	Rheumatoid arthritis
RA-ILD	Rheumatoid arthritis-associated interstitial lung disease
RREAL	Rapid Research, Evaluation and Appraisal
TYA	Teenage and young adult
