# Supplementary material for: Overcoming Patient Access Barriers in Complex Conditions: Lessons from Schizophrenia for Broader Healthcare Applications
Source: J Mark Access Health Policy. 2025 Dec 23;14(1):2. doi: 10.3390/jmahp14010002 (PMC12821714; doi:10.3390/jmahp14010002)
Supplement: Supplementary file 1 [file jmahp-14-00002-s001.zip › jmahp-3906026-supplementary.pdf]

Supplemental file

Semi-Structured Interview Guides Overview

This section provides the full semi-structured interview guides used for three participant groups: psychiatrists, other healthcare professionals (HCPs) and health policy experts (HPEs). The guides were developed based on targeted literature review to ensure comprehensive coverage of key topics, including patient access barriers, care pathway structures, reimbursement challenges and implementation issues. They were used to facilitate consistent, reproducible interviews while allowing flexibility to explore emerging insights.

Supplementary file S1. Interview discussion guide for Psychiatrists.

Purpose:
To explore treatment pathways, management of cognitive impairment associated with schizophrenia (CIAS), and reimbursement processes. This guide applies only to psychiatrist participants.

Research Objectives:

1. Validate the current care pathway for patients with schizophrenia.
2. Understand the current management of cognitive impairment in schizophrenia.
3. Explore potential management of cognitive impairment if a targeted drug were available.
4. Identify barriers and facilitators to establishing a care pathway for cognitive impairment in schizophrenia.
5. Explore reimbursement for schizophrenia care and implications for cognitive impairment.

Introduction (2 minutes)

- Introduce researcher and purpose of study.
- Explain confidentiality, recording, and adverse event reporting.
- Confirm participant consent to participate and to recording.

Warm-up and Schizophrenia Care (5 minutes)

Objective: Make the participant comfortable and learn about their general experience in managing schizophrenia.

Questions:

1. Describe your professional background and practice setting.
2. What region do you currently practice in?
3. How long have you worked in psychiatry?
4. How many patients with schizophrenia do you see per month on average?
5. At what point does your practice come into contact with patients, and what care do you provide?
6. How do you stay informed about advances in schizophrenia care (e.g., societies, research groups, CPD, conferences, literature)?

Part 1: Current Schizophrenia Pathway

Diagnosis and Treatment Initiation (10–12 minutes)

Objective: Understand first-episode psychosis diagnosis, treatment initiation, and prior risk assessment.

Questions:

1. Describe what happens when a patient experiences a first episode of psychosis in your region.
2. Who is the patient's first point of contact, and what is their role?
3. How does a patient come under your team's care?
4. Is there an early intervention service for psychosis? How long do patients receive treatment?
5. What does diagnosis involve? Who conducts and interprets assessments?
6. How is the treatment plan decided, and who is the prescriber?
7. What is the relative importance of pharmacological versus psychosocial interventions?
8. Are other drugs (apart from antipsychotics) prescribed?

9. Are patients ever assessed for risk before a first psychotic episode (e.g., UHR/ARMS)? What happens if they are deemed at risk?
10. (Optional) Describe the payment and reimbursement process at this stage. Who pays, reimburses, and what influences reimbursement?

<u>Ongoing Care (10 minutes)</u>

Objective: Understand ongoing management and reimbursement.

Questions:

1. What does ongoing care involve, and who is responsible?
2. What do you monitor (e.g., symptoms, medication response, psychosocial response, QoL, physical health)?
3. How frequently do you have contact with patients, and in what format (digital, phone, in-person)?
4. What happens if treatment needs adjustment, or if a patient relapses?
5. Are patients referred or transitioned to other levels of care (e.g., ACT, F-ACT)?
6. How is stability defined, and what support is provided at this point?
7. (Optional) Are there regional differences in care or protocols?
8. (Optional) Are there differences in payment or reimbursement at this stage, and are any treatments not reimbursed?

<u>Part 2: CIAS Care (30 minutes)</u>

<u>Existing CIAS Care (18 minutes)</u>

Objective: Explore current care and reimbursement for cognitive symptoms in schizophrenia.

Questions:

1. How aware are psychiatrists of cognitive symptoms in schizophrenia?
2. How are cognitive symptoms assessed, and who is involved?
3. What tools are used for assessment? Are assessments guideline-based?
4. What happens after assessment?

5. What pharmacological or psychosocial treatments are available? Who decides on initiation and delivers treatment?
6. How common is treatment use, and how are symptoms monitored?
7. How is care reimbursed (indications, requirements, coverage differences)?
8. Practice-specific: How many patients in the past month were assessed for cognitive symptoms? Describe a recent patient case (without identifying details).
9. For this case: How were cognitive impairments detected? What actions were taken? Who was involved in decision-making? What factors influenced decisions (clinical stage, medications, guidelines, reimbursement, patient context)?

<u>Hypothetical / Ideal CIAS Care (12 minutes)</u>

Objective: Explore barriers and facilitators for establishing an ideal CIAS pathway and assess interest in a targeted drug.

Questions:

1. In an ideal system, how should cognitive symptoms be identified and assessed (timing, team involvement, workflow)?
2. What currently prevents ideal identification or treatment (patient, HCP, system, reimbursement factors)?
3. What would facilitate ideal care?
4. Imagine a pharmacological product (Product X) for cognitive symptoms. How would its prescription, monitoring, and integration into care pathways work?
5. What impact could Product X have?
6. Are there other HCP roles, teams, or organizations influencing CIAS care pathways?
7. Final thoughts: Anything else the participant would like to add?

<u>Conclusion</u>

- Thank participant.
- Disclose sponsor (Boehringer Ingelheim).

Supplementary file S2. Interview discussion guide for other HCPs.

Purpose:
To explore the roles of various healthcare professionals (HCPs) in the treatment and management of schizophrenia, management of cognitive impairment associated with schizophrenia (CIAS), and reimbursement processes. This guide applies to non-psychiatrist HCP participants (e.g., GPs, nurses, psychologists, other specialists).

Research Objectives:

1. Validate the current care pathway for patients with schizophrenia.
2. Understand the current management of cognitive impairment in schizophrenia.
3. Explore potential management of cognitive impairment if a targeted drug were available.
4. Identify barriers and facilitators to establishing a care pathway for cognitive impairment in schizophrenia.
5. Explore reimbursement for schizophrenia care and implications for cognitive impairment.

Introduction (2 minutes)

- Introduce researcher and purpose of study.
- Explain confidentiality, recording, and adverse event reporting.
- Confirm participant consent to participate and to recording.
- Explain that the session involves a group discussion with multiple HCPs to explore their complementary roles.

Warm-up (5 minutes)

Objective: Help participants feel comfortable and learn about their specific roles.

Questions:

1. Introduce yourself, your role, and practice setting.
2. [GP only] How is a “special interest” in mental health defined in your practice setting?
3. What do you most enjoy about your job?

Part 1: Schizophrenia Pathway (15 minutes)

Objective: Understand participants' experiences managing and treating patients with schizophrenia, with emphasis on interprofessional collaboration.

Questions:

1. What role do you play in the care of patients with schizophrenia? What are your responsibilities?
2. At what point do you first come into contact with patients (stage of psychosis, risk assessment, diagnosis, treatment, ongoing care)?
3. Which teams do you work closely with?
    - Who refers to you?
    - Where does a patient go next?
    - Is there an early intervention for psychosis service in your region?
4. How do you communicate with other teams?
5. How do you communicate with patients (including digital tools)?

Part 2: CIAS Pathway – Current Care (20 minutes)

Objective: Explore current awareness, assessment, and management of cognitive symptoms in schizophrenia.

Questions:

1. At what points in care are cognitive symptoms considered in your practice?
2. What challenges exist in identifying or addressing cognitive symptoms?
3. How are cognitive symptoms currently identified?
    - What are the signs?
    - Are any tests or measures used? Which tests?
    - Who conducts the assessment? What is your role?
4. How are cognitive symptoms currently treated?
    - What pharmacological and psychosocial options exist?

- Who decides to initiate treatment, and what factors influence this decision?
- Who delivers treatment, and who monitors cognitive performance?

5. How much of a priority is addressing cognitive symptoms in schizophrenia for you and other HCPs in your role?
6. How many patients in the last month were assessed for cognitive symptoms, or could have had cognitive symptoms?

Part 3: CIAS Pathway – Hypothetical / Ideal Care (20 minutes)

Objective: Gather participants' perspectives on an ideal care pathway and potential targeted drug intervention.

Questions:

1. In an ideal system, how should cognitive symptoms be identified and assessed?
   - Timing of assessment
   - Which HCP or team should identify it
   - Assessment workflow and referrals
2. What prevents ideal identification or treatment in the current system?
   - Patient or caregiver factors
   - HCP factors
   - Health system factors
3. What would make the ideal scenario more likely to occur?
4. Imagine a pharmacological product (Product X) that targets cognitive symptoms (non-antipsychotic, used alongside antipsychotics).
   - Would you be interested in such a product? Why?
   - How would initiation or prescription work (timing, HCP roles)?
   - Who would monitor ongoing care?
   - How would it fit alongside psychosocial interventions?
   - Who would have ultimate responsibility for the patient?
5. In the current system, what could prevent this ideal pathway and what could facilitate it?

    - Patient/caregiver factors
    - HCP factors
    - Health system factors (e.g., reimbursement considerations)
6. (Optional if time) What impact could Product X have?
7. (Optional if time) Are there other HCP roles, teams, or organizations influencing CIAS care pathways?
8. Final thoughts: Anything else participants would like to add?

Conclusion

- Thank participants.
- Disclose sponsor (Boehringer Ingelheim).

Supplementary file S3. Interview discussion guide for HPEs.

<u>Purpose:</u>
To explore the reimbursement and funding pathways for schizophrenia care and cognitive impairment associated with schizophrenia (CIAS), including decision-making structures, access restrictions, and potential introduction of a new pharmacological treatment (Product X).

<u>Research Objectives:</u>

1. Understand the organization of care delivery systems (e.g., outpatient clinic, patient in home setting, patient in supported living).
2. Understand how care for CIAS is financed/funded and who pays in different settings.
3. Identify who decides or influences funding and access to CIAS care (committees, guidelines, protocols, etc.).
4. Explore factors influencing patient access to CIAS care (restrictions, requirements, limitations).

Introduction (2 minutes)

- Introduce researcher and purpose of study.
- Explain confidentiality, recording, and adverse event reporting.
- Confirm participant consent to participate and to recording.
- Explain that the session will explore current and hypothetical care scenarios in schizophrenia and CIAS.

Warm-up (3 minutes)

Objective: Make the participant feel comfortable and gather context on their experience.

Questions:

1. Please describe your background and current role.
   - How long have you been working in this area?
   - Who/which roles do you work with regularly?
   - What do you enjoy most about your work?
2. How do you typically gather information about pharmaceuticals entering the market?

Part 1: Delivery, Funding, and Reimbursement of Schizophrenia and CIAS Care (25 minutes)

Objective: Understand funding arrangements across different care models using patient case studies.

Case Study 1: Patrick

- Diagnosed with schizophrenia 7 years ago.
- Sees a psychiatrist every 3 months at an outpatient clinic.
- Positive symptoms managed with antipsychotics and CBT for psychosis (CBTp).
- Lives in supported accommodation with social worker support.
- Cognitive symptoms suspected to impair everyday functioning.

Questions:

1. How would funding for Patrick's antipsychotic medication be arranged?
    - Who decides eligibility for reimbursement (national/regional/local)?
    - Who pays for the medication (national/regional/local)?
    - What conditions or requirements exist for reimbursement (e.g., ICD-10/DSM codes, specialist referral, insurer approval)?
    - Are there limits or restrictions on reimbursement?
    - What factors could prevent or delay access, and how could the process be improved?
2. How would funding for CBT for psychosis be arranged?
    - Decision-making bodies and payment sources.
    - Requirements or conditions for reimbursement (e.g., number of sessions, diagnosis, disease stage).
    - Potential barriers or delays.
    - Opportunities for improvement.
3. How would funding for social care and support (e.g., social worker sessions) be arranged?
    - Decision-making and payment bodies.
    - Conditions or restrictions for reimbursement (e.g., functional assessments).
    - Potential barriers or delays.
    - Opportunities for improvement.
4. How would funding for cognitive testing be arranged if conducted by the outpatient clinic?
    - Decision-making and payment bodies.
    - Conditions for reimbursement.
    - Barriers or delays.
    - Opportunities for improvement.
5. If cognitive testing indicated impairment requiring care, how would this CIAS care be reimbursed?

- Conditions for reimbursement.
- Payment sources.
- Referral pathways in practice.

Case Study 2: Dominique

- Lives at home with her husband.
- Positive symptoms stable for over a year.
- Transitioning from specialist outpatient care to GP care.

Questions**:**

7. What are the funding implications of GP-led care for schizophrenia?
    - Decision-making bodies and payment sources.
    - Changes in reimbursement requirements compared with outpatient care.
    - If cognitive symptoms arise, how would screening and subsequent CIAS care be funded?
8. Are there other care delivery models relevant to cognitive health in schizophrenia?
    - How would funding for screening and treatment be arranged in these models?

<u>Part 2: Reimbursement for CIAS Care – Product X (30 minutes)</u>

Objective: Explore funding and reimbursement considerations for a hypothetical pharmacological treatment targeting CIAS.

<u>Product X Description:</u>

- Targets cognitive symptoms in schizophrenia.
- Non-antipsychotic, prescribed alongside antipsychotics.
- Oral administration, no therapeutic monitoring, does not replace cognitive remediation therapy.
- Expected benefits: improved cognition, functioning, quality of life, independence, and caregiver burden.

Questions:

1. Initial thoughts on pricing and reimbursement of Product X.

    - How would value and price be determined?

2. Who or which bodies would influence reimbursement decisions?
    - Governmental organisations, professional bodies, patient associations, care teams.
3. Who is involved in:
    - Recommendations for new treatments.
    - Setting guidelines for CIAS assessment and care.
    - Creating guidance where none exists.
4. What would need to change to ensure cognitive assessment and CIAS care occur in practice?
    - Key players and structures required.
    - Current barriers.
5. What factors influence reimbursement decisions?
    - Value for money, evidence requirements, prescribing pathways, reference product, patient acceptance, organizational/national policies.
6. What type of evidence would be accepted for Product X (clinical, patient experience, consensus statements)?
7. How could policy support cognition-focused care?
8. How could widespread clinical, social, or economic benefits be evidenced and valued?
9. Anticipated PICO (Population, Intervention, Comparator, Outcome) questions.
10. Potential challenges to reimbursement.
    - System, treatment, and patient factors.
11. Potential facilitators for reimbursement.
    - System, treatment, and patient factors.
12. Possible restrictions or conditions if Product X is reimbursed (e.g., indications, referral requirements, line of therapy).
13. Expected impact of Product X on CIAS care.
14. Role of payers in improving equitable access to care.

Conclusion

- Invite final comments or insights.
- Thank participants.
- Disclose sponsor (Boehringer Ingelheim)non-arteritic anterior ischemic optic neuropathy.

Supplementary Table S1. Participant inclusion criteria.

Participant Type	Inclusion Criteria	Notes
Psychiatrists	• 5–30 years of clinical experience<br>• Experience treating patients with schizophrenia<br>• Spend ≥50% of time treating patients<br>• Proficient in English	• Familiarity with cognitive impairment in schizophrenia: 2/3 with some familiarity, 1/3 with little to no familiarity<br>• Even distribution of gender, practice settings, and locations
Other HCPs (GP, clinical psychologist, psychiatric/mental health nurse)	• 5–30 years of experience<br>• Experience treating or working with patients with schizophrenia<br>• For GP, clinical psychologist, psychiatric/mental health nurse: spend ≥50% of time treating patients<br>• Proficient in English	• Even distribution of gender, practice settings, and locations<br>• GPs with "special interest or experience in mental health" defined as seeing a higher number of patients with mental health conditions, including severe conditions
HPEs	• ≥5 years of industry experience<br>• Not currently a public official<br>• Familiarity with reimbursement pathways for pharmacological and/or non-pharmacological treatments for severe mental illness<br>• Proficient in English	• Even distribution of gender, practice settings, and locations

Supplemental Table S2. Sociodemographic (A) and clinical (B) characteristics of HCP participants (n=32).

A) Sociodemographic characteristics

**Characteristic**		
Gender	Male	14
	Female	12
	Prefer not to say	1
	Unknown	5
Role	Psychiatrist	18
	Clinical psychologist	6
	Psychiatric nurse	2
	General practitioner	6

B) Clinical characteristics

**Characteristic**		**Psychiatrist (n=18)**	**Psychologist (n=6)**	**Psychiatric nurse (n=2)**	**GP (n=6)**	**Overall (n=32)**
Country of practice	Belgium	3	1	1	1	6
	Denmark	3	0	0	1	4
	Finland	2	2	0	0	4
	Greece	1	0	0	1	2
	Netherlands	3	1	0	0	4
	Norway	1	1	0	1	3
	Portugal	3	1	1	1	6
	Sweden	2	0	0	1	3
Years practicing specialty	5–14	8	0	0	2	10
	15–24	5	3	2	2	12
	≥25	3	0	0	2	5
	Unspecified (<5)	2	3	0	0	5
Time in direct patient care (%)	0–49	0	0	0	0	0
	50–74	4	1	0	0	5
	75–100	11	1	2	6	20
	Unknown	3	4	0	0	7
Practice setting*	Academic or teaching hospital	11	1	2	0	14
	District or general hospital	4	3	0	2	9
	Community-based	2	1	0	1	4
	Private	5	3	0	3	11

	Unknown	2	3	0	0	5
Practice location*	Urban	15	3	2	5	25
	Rural	0	1	0	1	2
	Unknown	3	3	0	0	6
Patients with SZ (n/year)	0–49	7	3	1	6	17
	50–99	1	0	0	0	1
	100–149	5	0	1	0	6
	≥150	3	0	0	0	0
	Unknown	2	3	0	0	5

SZ, schizophrenia.

*Participants were permitted to select multiple response options; therefore, percentages may exceed 100%.
